# Determining a threshold hospital size for the application of activity-based funding

**DOI:** 10.1186/1472-6963-11-S1-A10

**Published:** 2011-10-19

**Authors:** J Scuteri, L Fodero, J Pearse

**Affiliations:** 1 HealthConsult, Sydney, New South Wales, 2000, Australia; 2 Health Policy Analysis, Sydney , New South Wales, 2065, Australia

## Introduction

Under the Heads of Agreement – National Health Reform, reached at the Council of Australian Governments’ meeting on 13th February 2011, the Australian and State/Territory Governments agreed to establish a national approach to activity-based funding (ABF). They also agreed to fund, wherever possible from 1st July 2012, public hospitals on the basis of a national efficient price for each service provided to a public patient.

Clause 30 of that Agreement states that “some small rural hospitals will continue to be funded by block grants where ABF alone would not enable these hospitals to maintain community services obligations (CSOs)”. To move forward on ABF implementation, the Australian Government Department of Health and Ageing (DoHA) commissioned a project to determine which hospitals should be block-funded (that is, termed CSO hospitals).

## Methods

Based on a review of the relevant literature, in the context of implementation of ABF, a CSO was defined as:

“…a public hospital that, due to factors outside the control of local management, is unlikely to be financially viable under an activity based funding arrangement that reflects an efficient price set at the national or jurisdictional level.”

Once the definition was in place, the problem was then to identify the factors that are likely to result in a public hospital not being financially viable under ABF. The potential factors considered were volume of services; variability in acute-patient separations and bed days; number of DRGs with five or more acute patients per year; differences in the average cost per weighted separation; road distance to nearest regional hospital; and Remoteness Region of the Statistical Local Area in which the hospital is located.

These factors were chosen because they were potentially relevant, and also because they could be measured using available data. To assess the importance of the factors, potential CSO hospital profiles were constructed using data from national minimum data sets (NMDSs), as well as other sources, for the three most recently available years (2006/07 – 2008/09).

## Results

There were 427 smaller hospitals located in regional and remote areas assessed for CSO status. The data analysis produced clear evidence that ‘scale’ is the most important factor driving two of the key statistics that influence the financial viability of a hospital under ABF arrangements (these statistics being costs-per-episode and degree- of-variation in activity). Several measures of scale, including annual separations and bed-days, were tested and found to be correlated. After consideration, annual acute Casemix-adjusted separations was chosen as the scale measure, since it was also the principal grouping variable used to define existing hospital peer groups.

We then tackled the question of setting a scale threshold below which hospitals would be defined as CSO. Five approaches were used: examining the criteria employed to define existing peer groups; looking for discontinuities in the distribution of acute Casemix-adjusted separations across the 427 hospitals; modeling Casemix-based payments to determine how many hospitals might be disadvantaged by ABF; modeling the relationship between average costs and hospital scale; and considering self-reported CSO status. Across all factors, the analysis suggested that a CSO-hospital threshold of between 1,700 and 2,000 annual acute Casemix-adjusted separations was most suitable.

Although a scale threshold was determined, flexibility is required in interpreting the definition, since no mechanistic formula can appropriately reflect the circumstances that might apply to a hospital at a particular time. Also, it is recognized that there are problems with a definition that includes a scale measure based entirely on acute Casemix-adjusted separations. However, given the limitations of the existing data, it was not possible to consider a scale measure that incorporated activity levels for non-admitted and sub- / non-acute care services. These programs usually represent a significant portion of the services provided by small regional and rural hospitals, and a better definition of CSO hospitals would include these activities.

## Conclusions

Approximately 349 of the 427 facilities met the proposed definition of a CSO hospital. The key statistics for these hospitals show that the definition identifies a different group of hospitals from those not classified as CSOs. There will always be some debate at the boundary, but key statistics such as beds; staff numbers; admitted episodes; and even emergency-department, outpatient and community-health services numbers, show very significant scale differences.

Not surprisingly, there are also large differences in average cost and activity levels between CSO and non-CSO hospitals. Nonetheless, as national approaches to counting and costing of sub- / non-acute and non-admitted patient care services are agreed upon and implemented under ABF arrangements, the CSO definition and thresholds can be further improved.

